# Cultivar and Harvest Time of Almonds Affect Their Antioxidant and Nutritional Profile through Gut Microbiota Modifications

**DOI:** 10.3390/antiox13010084

**Published:** 2024-01-09

**Authors:** Adriana Delgado-Osorio, Beatriz Navajas-Porras, Sergio Pérez-Burillo, Daniel Hinojosa-Nogueira, Ángela Toledano-Marín, Silvia Pastoriza de la Cueva, Oleg Paliy, José Ángel Rufián-Henares

**Affiliations:** 1 Departamento de Nutrición y Bromatología, Instituto de Nutrición y Tecnología de los Alimentos, Centro de Investigación Biomédica, Universidad de Granada, Av. del Hospicio, s/n, 18012 Granada, Spain; adrianadelgado@ugr.es (A.D.-O.); beatriznavajas@ugr.es (B.N.-P.); spburillo@uma.es (S.P.-B.); dhinojosa@ugr.es (D.H.-N.); antolemarin@correo.ugr.es (Á.T.-M.); spdelacueva@ugr.es (S.P.d.l.C.); 2 Department of Biochemistry and Molecular Biology, Boonshoft School of Medicine, Wright State University, Dayton, OH 45435, USA; oleg.paliy@wright.edu; 3 Instituto de Investigación Biosanitaria ibs.GRANADA, Universidad de Granada, Avda. de Madrid 15, 2a Planta, 18012 Granada, Spain

**Keywords:** almond, cultivar, harvest time, digestion, fermentation, gut microbiota, metabolites

## Abstract

Almonds are a rich source of beneficial compounds for human health. In this work, we assessed the influence of almond cultivars and harvest time on their morphological (length, width and thickness) and nutritional (ash, moisture, proteins) profiles. We also evaluated the impact of an in vitro digestion and fermentation process on almonds’ antioxidant and phenolic content, as well as their support of gut microbiota community and functionality, including the production of short-chain fatty acids (SCFAs), lactic and succinic acids. The length, width, and thickness of almonds varied significantly among cultivars, with the latter two parameters also exhibiting significant changes over time. Moisture content decreased with maturity, while protein and ash increased significantly. Total antioxidant capacity released by almonds after digestion and fermentation had different trends depending on the antioxidant capacity method used. The fermentation step contributed more to the antioxidant capacity than the digestion step. Both cultivar and harvest time exerted a significant influence on the concentration of certain phenolic compounds, although the total content remained unaffected. Similarly, fecal microbiota modulation depended on the cultivar and maturity stage, with the Guara cultivar and late maturity showing the largest effects. Cultivar type also exerted a significant impact on the concentration of SCFAs, with the Guara cultivar displaying the highest total SCFAs concentration. Thus, we conclude that cultivar and harvest time are key factors in shaping the morphological and nutritional composition of almonds. In addition, taking into account all the results obtained, the Guara variety has the best nutritional profile.

## 1. Introduction

Almonds (*Prunus dulcis* (Mill.) D.A. Webb) are one of the most consumed nuts worldwide. These nuts are part of the Mediterranean diet and are eaten raw, blanched, roasted, fried, and caramelized; processed to flour; used in non-dairy beverages; or used as an ingredient for use in foods. In addition to their high gastronomic value, almonds stand out for their high nutritional profile. They represent an important source of lipids, mainly monounsaturated and polyunsaturated fatty acids, proteins, dietary fiber, and micronutrients such as vitamin E, potassium, phosphorus, calcium, and magnesium [1]. Almonds are also known to be a major source of phenolic compounds. These are responsible for the flavor and aroma of the almonds, as well as for their antioxidant, antiviral, and antibacterial properties [2,3].

The amount and type of nutrients present in almonds are influenced by different factors, including the cultivar, time of harvest, and the specific agroclimatic conditions in which they are grown [4]. Cultivar and harvest time are two of the main factors determining the morphological and nutritional profile of almonds. Numerous studies have confirmed the influence of these factors on morphological measurements (width, length, and thickness of the almond), as well as on the moisture, carbohydrate, lipid, protein, ash, antioxidant, mineral, phenolic, and tocopherol contents of almonds [5,6,7,8,9,10]. However, the results of these studies are partially contradictory, especially regarding the effect of harvest time, since there is no consistent trend on how the chemical profile of the almond evolves with time. These discrepancies could be related to the differences in those cultivars and harvest times considered, as well as due to other, yet unstudied, factors.

An additional key element in understanding how almond compounds impact human health is their bioaccessibility, which can be defined as the amount of a nutrient that is released from the food matrix in the gastrointestinal tract, and which becomes accessible for absorption. The bioaccessibility of nutrients is determined by multiple factors, including human digestion and fermentation by the gut microbiota [11]. In this context, dietary fiber and phenolic compounds are interesting to study because they are poorly digested in the upper gastrointestinal tract and enter the large intestine, where they are transformed by the gut microbiota into simpler metabolites with enhanced absorption and bioactivity [12]. The most common metabolites of dietary fiber, short-chain fatty acids (SCFAs), such as acetic, propionic and butyric acids, along with the metabolites of phenolic compounds have demonstrated multiple benefits in human health [13,14]. In addition to SCFAs, the gut microbiota produces other metabolites in the course of fermentation: lactic and succinic acids. These compounds have a significant impact on human health, as they are shared metabolites in human and microbial metabolic pathways and participate in microbiota–host cross-talk [15]. Other relevant actions of the gut microbiota include the release of antioxidant molecules from the food matrix and the production of antioxidant metabolites, leading to an increase in the antioxidant capacity of foods [16].

There is, however, insufficient information available on how human digestion and fermentation by the gut microbiota influence the antioxidant capacity of almonds and the bioaccessibility of their nutrients. A few studies have attempted to understand the effects of in vivo human digestion and fermentation by the gut microbiota on almonds, with a focus on analyzing gut microbiota-derived metabolites generated after the ingestion of almonds, such as the metabolites of phenolic compounds in urine and plasma [17,18,19] and the SCFAs in feces [20,21]. On the other hand, a group of studies focused on understanding the effects of in vitro digestion and fermentation and quantified SCFAs [22,23,24], phenolic compounds, and antioxidant capacity [24] generated after the process. Nevertheless, none of these studies took into account factors such as cultivar and harvest time, which could introduce potential variability in the results.

Thus, the aim of this work is to study the influence of harvest time and cultivar on morphological measurements (width, length and thickness of the almond) as well as the nutritional composition of almonds (ash, moisture and protein content) and evaluate the impact of an in vitro digestion and fermentation process in the antioxidant, phenolic, SCFAs, lactic and succinic acids profiles of almonds.

## 2. Materials and Methods

### 2.1. Chemicals

Firstly, (±)-6-Hydroxy-2,5,7,8-tetramethylchromane-2-carboxylic acid (Trolox), 2,2′-Azino-bis(3-ethylbenzothiazoline-6-sulfonic acid) diammonium salt (ABTS), 2,4,6-Tri(2-pyridyl)-s-triazine (TPTZ), 2,2 Diphenyl-1-1-picrythydrazul hydrate 95% (DPPH), potassium persulphate, 3,5-dicaffeoylquinic acid, caffeic acid, dimethyl caffeic, chlorogenic acid, ferulic acid, *p*-coumaric acid, gallic acid, tyrosol, *m*-hydroxyphenylacetic acid, Folin–Ciocalteu reagent, sodium hydroxide, methanol, hydrogen peroxide, hydrochloric acid, sulfuric acid, formic acid, succinic acid, propionic acid, acetic acid, isobutyric acid, lactic acid, (+)-catechin, 3-(3-hydroxyphenyl)propionic acid, 3,4-dihydroxyphenylacetic acid, 3-(3,4-dihydroxyphenyl)propionic acid, (-)-epicatechin, kaempferol, phenol, quercetin, *p*-coumaric acid, naringenin, phloroglucinol, ferulic acid, urolithin A, urolithin B, iron (III) chloride hexahydrate, sodium acetate, potassium chloride, potassium di-hydrogen phosphate, sodium mono-hydrogen carbonate, sodium chloride, magnesium chloride hexahydrate, ammonium carbonate, calcium chloride dihydrate, sodium di-hydrogen phosphate, tryptone, cysteine, sodium sulfide, resazurin, salivary α-amylase, pepsin, bile acids (porcine bile extract), ethanol and Milli-Q^®^ water were from Sigma-Aldrich. Pancreatin from porcine pancreas were purchased from Alpha Aesar. Diethyl ether and acetonitrile were from Honeywell. *N*-butyric acid was from Acros Organics, and 5-(3′, 4′-dihydroxyphenyl)-γ-valerolactone was purchased from TRC Canada. Moreover, (-)-Epigallocatechin and naringin were from Extrasynthese. Rutin was from PhytoLab. KjTabsTM VCM tablets, KjTabsTM VS Antifoam tablets, and boric acid solution (4% solution + indicator) were from Thermo Fisher Scientific (Waltham, MA, USA).

### 2.2. Samples

Almonds were cultivated in a private plot in Dúrcal (Granada, Spain, 36.962126, −3.560991). They belonged to the Spanish cultivars Guara (G), Vairo (V), Marta (MT), Marinada (MD), and Marcona (MC) and were harvested at three different times: time 1 (T1), time 2 (T2) and time 3 (T3). T1 and T2 for the five cultivars corresponded to 2/8/2023 and 24/8/2023, respectively, while T3 for Guara, Vairo and Marta cultivars corresponded to 26/9/2023 and T3 for Marinada and Marcona cultivars corresponded to 2/10/2023. After harvesting, the hulls and shells were removed, and almond kernels were stored at −80 °C until analysis.

### 2.3. Morphological Measurements

For each cultivar and harvest time, 10 almonds were randomly selected to measure kernel length, width and thickness by a caliper. Results were expressed in centimeters.

### 2.4. Determination of Ash, Moisture and Protein Content

Ash, moisture and protein content were determined in duplicate for each cultivar and harvest time by AOAC 923.03, 925.09 and 950.48 methods, respectively. Almond samples were grounded and homogenized, and 5 g of the mixture was used for each ash and moisture determination, while 0.5 g was employed for determining protein content. The results were expressed in percentages of ash, moisture, and protein content.

### 2.5. In Vitro Digestion and Fermentation

The samples were prepared by grinding each almond cultivar collected at each harvest time. Then, 100 mg of the resulting mixture was weighed. The samples were subjected to a previously described in vitro digestion and fermentation process [25] in order to simulate the human digestion–fermentation process without performing a nutritional intervention.

Briefly, the in vitro digestion consisted of an oral phase (2 min at 37 °C with 75 U/mL α-amylase), a gastric phase (2 h at 37 °C with 2000 U/mL pepsin at pH 3), and an intestinal phase (2 h at 37 °C with 13.37 mg/mL pancreatin and 10 mM bile acids at pH 7). The enzymatic reactions were stopped by immersing the tubes in ice. Once the three phases were finished, the samples were centrifuged at 6000 rpm for 10 min at 4 °C. The supernatant obtained represented the soluble and potentially absorbable fraction in the small intestine. Then, 10% of the supernatant was added to the solid residue, after which both mixed fractions were lyophilized and frozen at −80 °C. The remaining supernatant was also stored at −80 °C.

In vitro fermentation was performed with fresh feces from three healthy donors (people who had not taken antibiotics three months before the stool collection and with a body mass index within the 20–25 range). Stools were pooled to reduce inter-individual variability [26]. The sample submitted to fermentation was the combination of the solid residue obtained after in vitro digestion (100 mg) plus 10% of the digestion supernatant. Two control fermentations were run containing only fecal inoculum and buffer but no digested almonds (designated BL). In vitro fermentation took place at 37 °C for 24 h in an oxygen-depleted atmosphere. Upon completion, the samples were immersed in ice to stop microbial activity and centrifuged at 6000 rpm for 10 min. The supernatant, which represented the potentially absorbable fraction in the large intestine, as well as the pellet, were stored at −80 °C.

### 2.6. Antioxidant Assays

The antioxidant capacity was evaluated in the two fractions obtained after the in vitro digestion and fermentation of almonds: the supernatants obtained after digestion and fermentation. The assays were performed in duplicate for each sample. The sum of the two fractions accounts for the total antioxidant capacity that almonds can exert within the human body.

#### 2.6.1. Trolox Equivalent Antioxidant Capacity against ABTS Radicals (TEAC_ABTS_) Assay

TEAC_ABTS_ was tested following a previously described method [27]. Briefly, ABTS was prepared by mixing ABTS stock solution (7 mM) with 2.45 mM potassium persulphate and storing the mixture in the dark for 12 h, after which it was diluted with a 50:50 ethanol:water solution. Then, 280 µL of diluted ABTS and 20 µL of sample or Trolox standard were added to a transparent 96-well polystyrene microplate (Biogen Científica, Madrid, Spain) and absorbance readings at 730 nm were monitored for 20 min on a Cytation 5 microplate reader (Agilent Technologies, Santa Clara, CA, USA) at 37 °C. Calibration was performed with a Trolox stock solution ranging from 0.01 to 1.00 mg/mL. Results were expressed as mmol Trolox equivalents per kg of sample.

#### 2.6.2. Trolox Equivalent Antioxidant Capacity Referred to Reducing Capacity (TEAC_FRAP_) Assay

TEAC_FRAP_ was conducted following a previously described procedure [28]. The FRAP reagent was composed of 2.5 mL of 10 mM TPTZ solution, 2.5 mL of 20 mM FeCl_3_·6H_2_O and 25 mL of 0.3 M acetate buffer at pH 3.6. Moreover, 280 μL of FRAP reagent and 20 μL sample or Trolox standard were mixed in a 96-well microplate (Biogen Científica) and absorbance readings at 595 nm were monitored for 30 min with a Cytation 5 microplate reader (Agilent Technologies) at 37 °C. Calibration was performed with a Trolox stock solution ranging from 0.01 to 1.00 mg/mL. Results were expressed as mmol Trolox equivalents per kg of sample.

#### 2.6.3. Trolox Equivalent Antioxidant Capacity against DPPH Radicals (TEAC_DPPH_) Assay

TEAC_DPPH_ was performed as in [29]. Then, 20 μL of the sample was mixed with 280 μL DPPH reagent (74 mg DPPH/L methanol) in a transparent 96-well polystyrene microplate (Biogen Científica) plate. Absorbance readings at 517 nm were monitored for 60 min using a Cytation 5 microplate reader (Agilent Technologies) at 37 °C. Calibration was performed with a Trolox stock solution ranging from 0.01 to 1.00 mg/mL. Results were expressed as mmol Trolox equivalents per kg of sample.

#### 2.6.4. Folin–Ciocalteu Assay

For performing the Folin–Ciocalteu assay, the procedure described by [30] was adapted to a microplate reader. Furthermore, 30 μL of the sample was mixed in a well with 15 μL of Folin–Ciocalteu reagent and 255 μL of 2.35% sodium carbonate. Absorbance readings were monitored at 725 nm for 60 min at 37 °C in a Cytation 5 microplate reader (Agilent Technologies). Calibration was performed with a gallic acid stock solution ranging from 0.01 to 1.00 mg/mL. Results were expressed as mg of gallic acid equivalents/kg sample.

### 2.7. Ultra-High Performance Liquid Chromatography (UHPLC) Analysis

Chromatographic analyses were performed using a UHPLC Agilent Infinity II LC System equipped with a Diode Array Detector and a Refractive Index Detector.

#### 2.7.1. Analysis of Phenolic Compounds

Phenolic compounds were extracted from the fermentation supernatant with diethyl ether [31]. The extraction procedure was carried out in duplicate for each sample. The fermentation supernatant samples were centrifuged at 13,000 rpm for 2 min, after which 800 µL of the supernatant was taken. Subsequently, 1 mL of diethyl ether was added to the 800 μL of supernatant and stored at 4 °C in the dark for 24 h. After that time, the organic phase corresponding to the diethyl ether with the extracted phenolic compounds was collected in a new tube, and two new extractions were carried out with 1 mL of diethyl ether at room temperature. Subsequently, a rotary evaporator at 30 °C was used to evaporate the diethyl ether, and the dry residue was redissolved in 1 mL of Milli-Q water/methanol 1:1 (*v*/*v*). Finally, the extracts were filtered with a 0.22 µm filter and collected in vials.

For the UHPLC analysis, the column used was an Agilent Poroshell 120 SB-Aq (4.6 × 100 mm, 2.7 µm). The mobile phase consisted of Solvent A (Milli-Q water/formic acid, 99.9:0.1) and Solvent B (acetonitrile/formic acid, 99.9:0.1). The method used was a gradient elution: 0–28 min (20% Solvent A, 80% Solvent B), 28–32 min (60% Solvent A, 40% Solvent B), 32–33 min (95% Solvent A, 5% Solvent B), 33–35 min (20% Solvent A, 80% Solvent B), 35–38 min (95% Solvent A, 5% Solvent B). The flow rate was maintained at 0.2 mL/min. The injection volume was 5 µL. The column temperature was set at 30 °C. The wavelength selected to measure the absorbance of the samples was 255 nm. The chromatographic analysis was performed in duplicate for each sample.

Quantification was carried out using calibration with the following external standards: (+)-catechin, (-)-epicatechin, (-)-epigallocatechin, rutin, 3,4-dihydroxyphenylacetic acid, 3-(3,4-dihydroxyphenyl)propionic acid, 3-(3-hydroxyphenyl)propionic acid, 5-(3′,4′-dihydroxyphenyl)-γ-valerolactone, phenol, phloroglucinol, ferulic acid, *p*-coumaric acid, naringenin, naringin, urolithin A, urolithin B, quercetin and kaempferol. Results were expressed in mg phenolic compound/kg of digestion residue.

#### 2.7.2. Analysis of SCFAs, Lactic and Succinic Acids

The preparation of the sample for the chromatographic analysis was performed by centrifuging the fermentation supernatant samples at 13,300 rpm for 5 min, filtering them through a 0.22 μm filter and performing a 1:10 dilution with 1 M hydrochloric acid [32]. The column used was an Agilent Poroshell 120 SB-Aq (3 × 150 mm, 2.7 μm). The mobile phase was 5 mM sulfuric acid with isocratic elution at a flow rate of 0.5 mL/min. The injection volume was 5 µL. The column and RID temperature were set at 35 °C. The chromatographic analysis was performed in duplicate for each sample.

Quantification was carried out using calibration with the following external standards: lactic, acetic, succinic, propionic, *N*-butyric and isobutyric acids. *N*-butyric and isobutyric acids were quantified together. Total SCFAs were calculated as the sum of acetic, propionic and butyric acid. Results were expressed in mmol of each acid per L of the fermentation supernatant.

### 2.8. Microbial Genomic DNA Isolation and High Throughput Sequencing

Prokaryotic genomic DNA was isolated from the pellet obtained after sample fermentation using a ZR bacterial/fungal DNA kit (Zymo Research, Irvine, CA, USA) following the manufacturer’s instructions. The V4 hypervariable region of the 16S rRNA gene was amplified using primers complementary to the flanking conserved sequences (forward primer complementary sequence GCCAGCMGCCGCGG and reverse primer complementary sequence GGACTACHVGGGTWTCTAAT). The forward primer incorporated a 6–7 nucleotide barcode to allow for sample multiplexing on the sequencer. In PCR amplifications, 25 ng of the starting gDNA material was first subjected to four cycles of linear elongation with the forward primer only in order to reduce sample-to-sample PCR bias [33], followed by twenty-five cycles of traditional exponential PCR. Amplicon sequencing was carried out on the Ion Torrent Personal Genome Machine (Thermo Fisher, USA) using a 318 Chip v2. After quality filtering, we obtained 32,485 reads per sample on average. All sequence reads were processed in QIIME [34] following our standard pipeline [35] to obtain the 16S rRNA gene-copy, number-adjusted, rarefied taxon counts. This final dataset was used for all further analyses.

### 2.9. Statistical Analyses

The statistical significance of the data was tested by the non-parametric Kruskal–Wallis analysis of variance test, followed by the pair-wise Games–Howell post-hoc tests to compare the samples that showed significant variation (*p* < 0.05). The harvest time and cultivar of the almonds were used as factors in the Kruskal–Wallis tests. All statistical analyses were performed using SPSS.

Multivariate statistical analyses were performed on the genus-level microbial abundance dataset generally following the approaches we described previously [36]. These included unconstrained principal coordinates analysis (PCoA) utilizing phylogenetic weighted UniFrac distance as a measure of sample dissimilarity and constrained canonical correspondence analysis (CCA). Logistic regression (LR) with Lasso regularization (threshold C = 0.2) was chosen to generate sample classification models, as was the case previously [37]. Lasso regularization allowed us to limit the number of discriminatory variables defining each sample type. Model performance was assessed by a 20-fold cross-validation algorithm. Statistical tests (one-way analysis of variance (ANOVA) unless otherwise stated) were carried out in SPSS.

## 3. Results and Discussion

### 3.1. Influence of Cultivar and Harvest Time on Ash, Moisture, Protein, and Morphology of Almonds

The harvest time of almonds had a significant impact on their width and thickness, while their length remained unaffected (Supplementary Materials, Table S1). Almond thickness tended to decrease significantly over time, while almond width initially increased and then returned to its original level. This general decrease can be attributed to a loss of moisture over time. However, the evolution of morphological measurements over time did not follow the same trend for all cultivars (Table 1). In fact, when comparing the morphological measurements between the five cultivars, significant differences were observed in all three morphological measurements (Supplementary Materials, Table S2). These results reinforce the idea that cultivar plays a major role in almond morphology.

A study conducted on 24 traditional almond cultivars in the central–western Iberian Peninsula revealed differences in the width, thickness, and length of these cultivars [38]. Another study involving 10 cultivars of diverse origins reported variations in the morphological measurements among them and highlighted distinct trends in almond length and width over time for each one [10]. In the same study, over the course of the two harvest times considered, almond length increased in five cultivars while decreasing in the other five. However, almond width increased in seven cultivars while decreasing in three. Additionally, the thickness decreased over time for the 10 cultivars under examination [10]. Thus, genotype has a strong influence on almond morphological characteristics.

In terms of the chemical composition, studied cultivars did not elicit a significant impact on the moisture, protein or ash content (Supplementary Materials, Table S3). Nevertheless, the cultivars with the highest levels of protein and ash were Vairo and Guara, respectively, while the lowest moisture was found in the Vairo cultivar.

For all cultivars, moisture content decreased significantly over time (Table 1), consistent with the previous reports [10,39,40,41,42,43]. A higher moisture level shortens the final product shelf life. We observed a significant increase in protein and ash content over time (Supplementary Materials, Table S4). There were significant differences between the first and the third harvest times: the ash content tripled, and the protein content doubled probably due to the decrease in water content. Regarding ash content, all five cultivars followed a consistent trend of increasing their content at each harvest time. However, in terms of protein content, all cultivars increased from T1 to T3, with Vairo and Marta cultivars experiencing a slight decrease from T1 to T2. These trends suggest that almonds accumulate protein and minerals over time, resulting in an increase in their nutritional value. The increase in the protein [10,43,44] and ash [10] content has been reported in the literature. However, although ash content serves as an indicator of mineral content, the trend in mineral levels can vary depending on the specific mineral being analyzed [6] and the almond cultivar [7,8,45].

In summary, these results indicate that the optimal time to harvest almonds is during the third period, as it leads to increased nutritional value and reduced moisture content (Supplementary Materials, Table S4), which in turn extends shelf life.

### 3.2. Antioxidant Capacity of the Samples Obtained after In Vitro Digestion and Fermentation

#### 3.2.1. Evolution of the Total Antioxidant Capacity over Harvest Time

**Figure 1:**
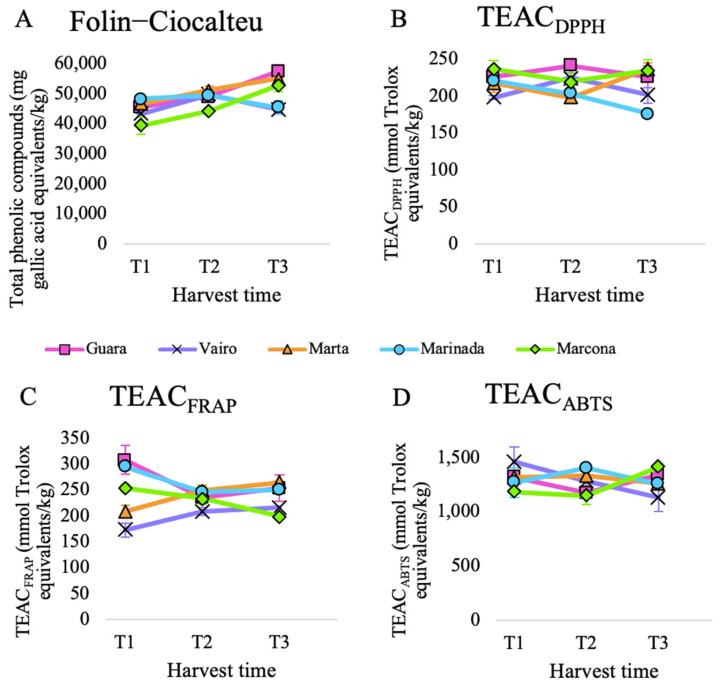
Total antioxidant capacity of five almond cultivars collected at three harvest times and submitted to in vitro digestion-fermentation measured by (**A**) Folin–Ciocalteu, (**B**) TEAC_DPPH_, (**C**) TEAC_FRAP_ and (**D**) TEAC_ABTS_ assays.

Total antioxidant capacity was calculated by adding antioxidant capacity released during in vitro digestion and in vitro fermentation. The evolution of total antioxidant capacity over harvest time differed among the assays (Figure 1).

The total phenolic content, measured via the Folin–Ciocalteu assay, exhibited significant differences over time, increasing at each time point, except for the Marinada cultivar (Supplementary Materials, Table S5) (Figure 1A). This general increase can be attributed to the accumulation of phenolic compounds during almond development. A study that analyzed the total phenolic content of almonds harvested at two different times also reported an increase for six out of the ten evaluated cultivars [10].

When analyzing the evolution of TEAC_ABTS_, TEAC_FRAP_, and TEAC_DPPH_ over time, no significant differences were observed (see Supplementary Materials, Table S5). The general trend for TEAC_DPPH_ was a decrease over time, observed in all cultivars from T1 to T3 except for Marta (Figure 1B). This contrasts with a study that reported an increase in TEAC_DPPH_ over time for 9 out of the 10 cultivars [10].

On the other hand, the general tendency for TEAC_FRAP_ and TEAC_ABTS_ was an initial decrease followed by a slight increase (Supplementary Materials, Table S5). This phenomenon could be attributed to the fact that TEAC_FRAP_ and TEAC_ABTS_ tend to yield comparable results because the same compounds are reactive in the FRAP and ABTS assays. However, there was no common pattern in the evolution of TEAC_FRAP_ and TEAC_ABTS_ when considering different cultivars. While cultivars Marta and Vairo tended to increase their TEAC_FRAP_ from T1 to T3, Guara, Marinada, and Marcona did the opposite (Figure 1C). Regarding TEAC_ABTS_, Marinada, Marta and Vairo tended to suffer a decrease in antioxidant capacity from T1 to T3, while Guara and Marcona showed an increase (Figure 1D).

When we compared cultivars against each other, we observed significant differences among cultivars for TEAC_FRAP_ and TEAC_DPPH_ (see Supplementary Materials, Table S6). For FRAP and DPPH assays, Guara was the cultivar that exhibited the highest total antioxidant capacity. For ABTS, it was Marinada, and for Folin–Ciocalteu, it was Marta. Different studies from different regions in the world have analyzed the influence of the cultivar on the antioxidant capacity of almonds [46,47,48]. However, not many studies have focused on Spanish cultivars. In one study [10], which included the Spanish cultivar Marcona, featured in this experiment, DPPH and Folin–Ciocalteu assays showed a wide variability among the 10 almond cultivars studied, highlighting the substantial influence of almond genotype on the antioxidant capacity of almonds. In this study, Marcona was one of the cultivars that exhibited lower values in both assays, as was the case in our study for Folin–Ciocalteu but not for TEAC_DPPH_. In another study that analyzed almond skin extracts [49], Guara reported a higher TEAC_FRAP_ and total phenolic content than Marcona, while Marcona had higher TEAC_DPPH_ values than Guara [50]. Nevertheless, Marcona almond oil was reported to have higher TEAC_DPPH_ than Guara almond oil.

Altogether, these results highlight the notorious impact of the cultivar as well as the antioxidant assay used to measure the evolution of the total antioxidant capacity over time.

#### 3.2.2. Contribution of In Vitro Digestion-Fermentation Fractions to Total Antioxidant Capacity

**Figure 2:**
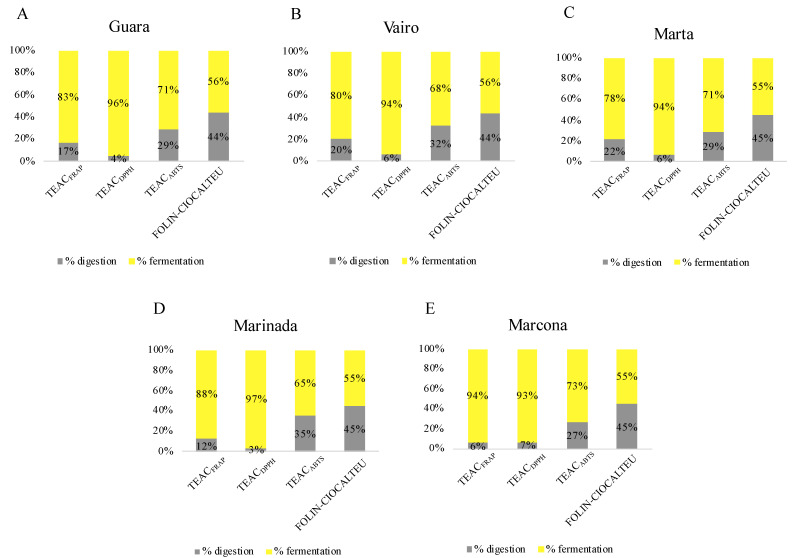
Contribution of the in vitro digestion and fermentation fractions to the total antioxidant capacity of (**A**) Guara (**B**) Vairo (**C**) Marta (**D**) Marinada (**E**) Marcona almonds harvested at T3.

The contribution of each fraction to the total antioxidant capacity is shown as a percentage in Figure 2. Data from almonds harvested at T3 were used for this purpose since it is considered the ideal maturity for consumption, as stated above. Fermentation released higher antioxidant capacity than in vitro digestion in all four assays used (Figure 2). This contribution was most notable in the DPPH assay, followed by the FRAP, ABTS and Folin–Ciocalteu assays. This pattern has also been seen in other studies. According to Li et al. [24], the fermentation fraction exhibited higher TEAC_DPPH_, TEAC_FRAP_ and total phenolic content measured by Folin–Ciocalteu than oral, gastric, and intestinal digestion fractions. That study also found that, in general, colonic fermentation provided the highest bioaccessibility for most phenolic compounds, thereby contributing to the antioxidant capacity. In another investigation of almond bagasse [51], fermentation contributed more to TEAC_DPPH_ and total phenolic content measured by Folin–Ciocalteu than gastric and intestinal digestions. In another study on nuts, the fermented fraction contributed more to TEAC_DPPH_, TEAC_FRAP_ and total phenolic content measured by the Folin–Ciocalteu assay in comparison with the digested fraction. This may be attributed to the gut microbiota releasing antioxidant compounds from the food matrix or generating antioxidant metabolites from compounds that have undergone incomplete digestion and absorption in the upper gastrointestinal tract. Almonds are a rich source of dietary fiber [23], which decreases the bioaccessibility of some nutrients during digestion and allows them to reach the large intestine [52]. There, the gut microbiota increases the bioaccessibility of these compounds as well as releases metabolites that contribute to the antioxidant capacity of the almond.

### 3.3. Microbiota Community Structure Supported by Fermentation of Digested Almond Samples

**Figure 3:**
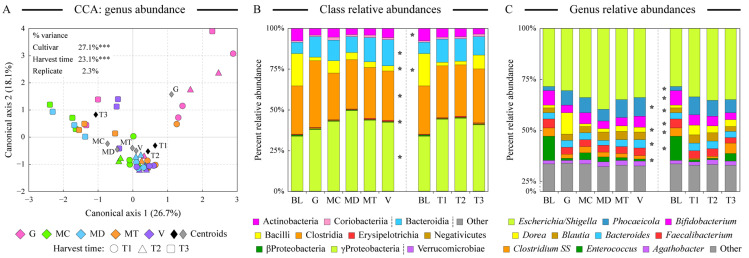
Comparison of microbiota composition among fermented samples. Similarity of microbial communities at the genus taxonomic level among all almond-fermented samples was assessed by the constrained canonical correspondence analysis (CCA, panel (**A**)). The percentage of total dataset variance explained by each axis is shown in parentheses. The relative contribution of explanatory variables to the overall variance of the dataset is shown with *** denoting *p* < 0.001. Panels (**B**,**C**) display the relative microbial abundances among sample types at the class (panel (**B**)) and genus (panel (**C**)) levels. Classes are ordered based on their phylum assignment; genera are ordered by the average abundance among all samples. Note the compression of the Y axis between 0% and 25% relative abundance values in panel (**C**). Star denotes the statistically significant difference (at α = 0.01 level) in the taxon abundance among sample groups as calculated by the analysis of variance algorithm. Abbreviations are as follows: G (Guara), MC (Marcona), MD (Marinada), MT (Marta), V (Vairo), BL (fermentation blank).

We used the 16S rRNA gene amplicon sequencing to obtain profiles of human fecal microbial communities maintained on the digested almond samples. Ordination analyses indicated that the microbiota community structure overlapped among different cultivars and harvest time samples with few notable exceptions (Figure 3A). The microbiota community supported by the fermentation of the Guara almond cultivar was the most distinct among cultivars; many of the late-harvesting-time (T3) samples also clustered together. Both the cultivar and the harvest time had statistically significant contributions to the overall microbiota abundance dataset variance, as revealed by the constrained CCA analysis (Figure 3A). All communities were dominated by the genus *Escherichia*/*Shigella* in class Gammaproteobacteria, likely indicating the presence of residual oxygen in the fermentation vessels. An abundance of the genus *Phocaeicola* (formerly members of the Bacteroides genus, class Bacteroidia) was supported by all almond samples in comparison with blanks. Blank samples, on the other hand, maintained the genus *Enteroccus*. Examining the distribution of microbial classes and abundant genera among sample types, Guara almond fermentation expanded the abundance of the genus *Dorea* (see Figure 3B,C), known for its ability to degrade dietary fiber [53]. Considering differences among various harvesting times, T3 samples were significantly more abundant in the microbial phylotypes assigned to *Clostridium sensus stricto* (*SS*). Earlier harvesting time promoted a higher abundance of *Blautia* members (Figure 3C). Despite the presence of large amounts of unsaturated fatty acids in almond seeds, we have not detected appreciable amounts of previously noted “lipophilic” microbial genera such as *Bilophila* and *Alistipes* [54] in the fermented almond samples. The abundance of Gammaproteobacteria, another “lipophilic” taxon, did increase in the presence of digested almonds (Figure 3B).

**Figure 4:**
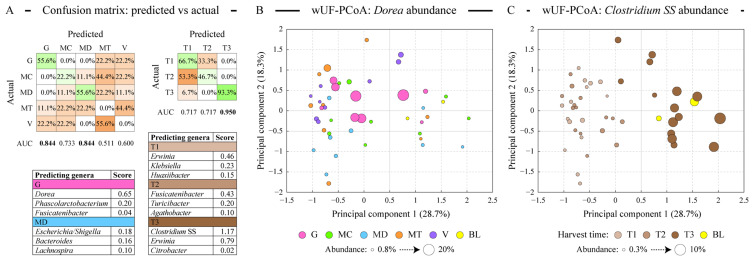
Modeling the differences in the microbiota profiles among sample types. Panel (**A**) displays the results of logistic regression (LR) based discriminant analysis of sample types. Separate models were generated for the microbiota distribution among the almond variety sample types and among the almond maturity sample types as shown. Confusion matrices reveal the concordance of the predicted vs actual class labels of all profiled samples, displayed as proportions of actual class labels assigned by the LR classifier to different classes. Green background highlights the correct prediction and orange background—misclassified cases. Area under the curve (AUC) values (range 0–1) illustrate the performance of each LR classifier. The top three genera predicting sample classes with high AUC values are shown in the tables; higher score represents higher contribution of the relative abundance of that genus to the classification model. Panels (**B**,**C**) depict the distribution of all samples in the weighted UniFrac distance-based principal coordinates analysis (PCoA). The percentage of total dataset variance explained by each axis is shown in parentheses. The size of each circle is proportionate to either *Dorea* (panel (**B**)) or *Clostridium sensus stricto* (SS, panel (**C**)) relative abundance in the corresponding sample as shown in the panel legend. Abbreviations are as follows: G (Guara), MC (Marcona), MD (Marinada), MT (Marta), V (Vairo), BL (fermentation blank).

We used a logistic regression algorithm to determine whether microbial communities can be classified into distinct classes based on either almond cultivar or harvest time. The classification procedure generated models predicting the classification of each sample based on its microbial composition, and the outcome of this analysis is shown in Figure 4A. Consistent with ordination analysis described above, the Guara cultivar together with Marinada was predicted the best among cultivars. Among harvest time classes, the late-harvested samples (T3) were the only class with great classification (93.3% correct prediction’ area under the curve score of 0.950). Due to the applied Lasso regularization, only a handful of microbial genera designated each class, as displayed in the lists in Figure 4A. The abundance of *Dorea*, which defined the microbiota of the fermented Guara cultivar, is highlighted on the weighted UniFrac-based PCoA plot shown in Figure 4B. Similarly, *Clostridium SS* abundance, defining the T3 sample type, is overlaid onto the same PCoA plot in Figure 4C. The abundance of the genus *Erwinia* (class Gammaproteobacteria) was found to differ among all three harvest types—it had an average relative abundance of 0.61% in T1 samples; 0.48% abundance in T2 samples, and 0.14% in T3 samples.

### 3.4. Phenolic Compounds, SCFAs and Lactic and Succinic Acids Measured after In Vitro Digestion and Fermentation

#### 3.4.1. Phenolic Compounds

In the analysis of the influence of the cultivar on the concentrations of individual phenolic compounds (Table 2), we observed significant differences among the cultivars for the concentration of (-)-epicatechin, (-)-epigallocatechin, ferulic acid, *p*-coumaric acid, rutin, 3-(3,4-dihydroxyphenyl)propionic acid, 3-(3-hydroxyphenyl)propionic acid, 5-(3′,4′-dihydroxyphenyl)-γ-valerolactone, phloroglucinol and urolithin B. The total concentration of individual phenolic compounds did not vary significantly among cultivars, similar to our results for the total phenolic content measured by the Folin–Ciocalteu assay. However, it is worth noting that the cultivar with the highest phenolic compound concentration was Guara. Other studies have reported variations in the individual profile of phenolic compounds among cultivars, with the extent of variation depending on the almond’s origin [47]. In Spanish cultivars, Marcona and Guara almond skin extracts reported differences regarding individual phenolic concentrations. Guara had higher concentrations of (+)-catechin, (-)-epicatechin and naringin compared to Marcona, while Marcona had higher concentrations of naringenin than Guara [50].

From the 18 phenolic compounds quantified in our study, 10 were phenolic compounds naturally found in almonds. Their order of abundance was, from highest to lowest: (+)-catechin, rutin, (-)-epicatechin, quercetin, (-)-epigallocatechin, kaempferol, naringenin, *p*-coumaric acid, ferulic acid, and naringin. The order of abundance of these phenolic compounds in almonds is similar to that reported in a review that brings together data from 61 studies [2]. Our results are generally within the ranges reported in that review, although we found some of the compounds at higher concentrations. This could be attributed to the increase in the bioaccessibility of phenolic compounds during in vitro fermentation, since a large proportion of these phenolic compounds are associated with dietary fiber, which remains intact after digestion and becomes a substrate for the gut microbiota that releases the bound phenolic compounds.

Harvest time did not have a significant impact on the concentration of 13 out of the 18 phenolic compounds analyzed (Table 3). Nevertheless, the concentrations of (-)-epigallocatechin, quercetin, and phenol exhibited a significant decrease over time, while the concentration of naringenin significantly increased. The total concentration of individual phenolic compounds showed no significant variation among harvest times but displayed a tendency to decrease. From T1 to T3, 13 phenolic compounds decreased their content, while 3 of them increased and 2 remained stable. The decrease in phenolic compound concentrations over time does not align with the significant increase observed in the total phenolic content determined by the Folin–Ciocalteu assay. This discrepancy may arise from factors such as not measuring the concentrations of all almond phenolic compounds or the reactivity of the Folin–Ciocalteu reagent with non-phenolic reducing compounds [55].

To the best of our knowledge, there are no studies evaluating the evolution of the individual phenolic profile of almonds during maturation. For other fruits, such as figs, the concentration of individual phenolic compounds decreased from T1 to T3 for 6 out of the 10 compounds determined [56]. In another study [57], the total concentration of individual phenolics decreased in various apple cultivars during ripening, although the trends were not consistent across all cultivars. Furthermore, it has been demonstrated that some in vitro functional properties of vegetables vary with harvest time, with hops showing higher phenolic content and functional properties in the early harvests due to modifications in the profile of phenolic compounds [58].

We also identified eight microbial phenolic metabolites, listed in order from highest to lowest concentrations: 5-(3′,4′)-dihydroxyphenyl-γ-valerolactone, phloroglucinol, 3-(3,4-dihydroxyphenyl)propionic acid, 3,4-dihydroxyphenylacetic acid, 3-(3-hydroxyphenyl)propionic acid, phenol, urolithin A, and urolithin B. This follows the trend of abundance reported by a clinical study [19] that measured phenolic microbial metabolites in urine after the ingestion of an almond skin extract. The most abundant metabolite, 5-(3′,4′-dihydroxyphenyl)-γ-valerolactone, could be a marker of the transformation of almond flavan-3-ols and proanthocyanidins. Moreover, 3-(3,4-Dihydroxyphenyl)propionic acid, 3-(3-hydroxyphenyl)propionic acid, and 3,4-dihydroxyphenylacetic acid are also metabolites of flavan-3-ols, as well as of flavonols, flavones, flavanones, and hydroxycinnamic acids. Phloroglucinol is a metabolite arising from the metabolization by the gut microbiota of a diverse group of phenolic compounds, among which we find flavonols, flavones, flavanones, flavan-3-ols, isoflavones, and anthocyanins. Phenol is one of the simplest metabolites that can be derived from the degradation of the parent phenolic compounds by the intestinal microbiota. Urolithins A and B are produced by the transformation of ellagic acid, a compound present in ellagitannins [59,60].

#### 3.4.2. SCFAs, Lactic and Succinic Acids

We observed significant differences in propionic, butyric, and succinic acid content among maturation stages, with their levels increasing from T1 to T3 (Table 4). The amount of acetic and lactic acids also increased from T1 to T3, but the differences were not statistically significant. Furthermore, the total SCFA content did not vary significantly among harvest times, although there was a slight increase as nuts matured. This suggests that T3 is the optimal time to harvest almonds, as it produces the highest SCFA concentrations upon gut microbiota fermentation. This could be attributed to T3 being the time when almonds have accumulated the highest fiber content (probably due to moisture loss), serving as a substrate for SCFA production. It has been demonstrated that almonds are a rich source of total dietary fiber and soluble dietary fiber [23], and that levels of almond neutral detergent fiber increase with fruit development [43]. These results could explain the increase in total SCFAs over time seen in our study.

We also detected significant differences when comparing SCFA production among cultivars (Table 4). Vairo and Marcona cultivars had the highest content of lactic and succinic acids, respectively. In contrast, Guara exhibited the highest total SCFAs and acetic, propionic, and butyric acid levels. This could be attributed to Guara having the highest fiber content, matching the results of supported microbiota community analysis described above. In a study comparing three apple cultivars [61], the cultivar with the highest total and soluble dietary fiber content reported the highest total SCFAs compared to the rest. These findings suggest that Guara might be the best cultivar choice due to its higher proportion of beneficial SCFAs and lower lactic and succinic acid levels, which are intermediates in metabolic pathways and whose effects on health are not yet fully understood.

To the best of our knowledge, no studies have evaluated the production of SCFAs and lactic and succinic acids following the in vitro digestion and fermentation of almonds for the purpose of comparing different cultivars and harvest times. The studies available in the literature primarily focus on whether almond consumption in vitro or in vivo could be beneficial in terms of SCFA production. Previous studies on the in vitro digestion and fermentation of almonds have assessed SCFA production using different approaches. Some studies have compared the SCFA production of various nuts and have consistently ranked almonds as a significant source, placing them second out of five [62], first out of five [63], first out of six [24] and third out of five [23] among the nuts studied. Another study observed a substantial production of butyrate following an in vitro digestion and fermentation of finely ground almonds, which could be attributed to the disruption of cell wall fibers and increased bioaccessibility [22]. On the other hand, in vivo studies have not found substantial differences after the consumption of almonds in terms of SCFA production [20,21]. However, there is a need for more clinical studies to further explore the benefits of almond consumption [64].

## 4. Conclusions

This work confirms the influence of the cultivar and harvest time on some morphological and nutritional characteristics of almonds. Cultivar type had a significant impact on length, width and thickness, but not on moisture, protein and ash content. Width, thickness and moisture content significantly decreased over time, while the protein and ash increased. The total antioxidant capacity released by almonds after an in vitro digestion and fermentation process had different trends: total phenolic content measured by the Folin–Ciocalteu assay significantly increased over time, while TEAC_ABTS_, TEAC_FRAP_ and TEAC_DPPH_ remained unaffected. Nevertheless, we reported significant differences for TEAC_FRAP_ and TEAC_DPPH_ among cultivars. The fermentation step contributed more to the total antioxidant capacity of almonds than the digestion step. The concentration of individual phenolic compounds was influenced more significantly by cultivar type than by harvest time. The same behavior was observed with total SCFAs. Nevertheless, neither cultivar nor harvest time had a significant impact on the total concentration of individual phenolic compounds.

The Guara cultivar showed the best nutritional profile, primarily due to its high levels of ash, protein, total SCFAs, TEAC_FRAP_, TEAC_DPPH_, total phenolic content measured by the Folin–Ciocalteu assay, and total concentration of individual phenolic compounds. In addition, the best harvest time was T3, characterized by a higher total phenolic content measured by the Folin–Ciocalteu assay along with increased ash, protein, total SCFAs, and reduced moisture. Consistent with these findings, the Guara cultivar and T3 harvest time supported human-derived gut microbial communities distinct from the rest of the almond samples.

## Figures and Tables

**Table 1:** Morphological measurements, ash, moisture and protein profile of five almond cultivars collected at three harvest times and submitted to in vitro digestion-fermentation.

Cultivar	Harvest Time	Length (cm)	Width (cm)	Thickness (cm)	Ash (%)	Moisture (%)	Protein (%)
Guara	T1	2.47 ± 0.13	1.09 ± 0.10 ^a^	0.70 ± 0.09 ^a^	1.38 ± 0.12 ^a^	65.1 ± 1.10	11.2 ± 1.13 ^a^
	T2	2.51 ± 0.13	1.28 ± 0.08	0.70 ± 0.05 ^a^	1.40 ± 0.21 ^a^	47.9 ± 0.20	12.8 ± 0.62 ^a^
	T3	2.41 ± 0.06	1.14 ± 0.14 ^a^	0.40 ± 0.05	5.55 ± 0.27	7.00 ± 0.10	24.6 ± 4.82
Significance		NS	*	*	NS	*	*
Vairo	T1	2.24 ± 0.09	1.04 ± 0.09	0.56 ± 0.07 ^a^	1.61 ± 0.01 ^ab^	63.1 ± 0.6	14.8 ± 5.54 ^ab^
	T2	2.18 ± 0.11	1.11 ± 0.07	0.55 ± 0.05 ^a^	1.65 ± 0.14 ^a^	44.7 ± 0.8	13.8 ± 1.03 ^a^
	T3	2.26 ± 0.10	1.11 ± 0.12	0.36 ± 0.05	3.63 ± 0.25 ^b^	6.00 ± 0.30	22.3 ± 0.72 ^b^
Significance		NS	NS	*	NS	*	*
Marta	T1	2.34 ± 0.13	1.01 ± 0.12 ^ab^	0.73 ± 0.05 ^a^	1.37 ± 0.06	66.9 ± 1.1 ^a^	12.5 ± 3.28
	T2	2.27 ± 0.12	1.10 ± 0.07 ^a^	0.71 ± 0.07 ^a^	1.64 ± 0.04	55.1 ± 2.00 ^a^	9.36 ± 2.56
	T3	2.25 ± 0.12	0.93 ± 0.09 ^b^	0.42 ± 0.06	4.56 ± 0.69	6.50 ± 0.00	22.6 ± 1.13
Significance		NS	*	*	NS	NS	*
Marinada	T1	2.07 ± 0.12 ^a^	1.09 ± 0.10 ^ab^	0.71 ± 0.03 ^a^	1.14 ± 0.07	76.6 ± 0.30	6.96 ± 0.21
	T2	2.28 ± 0.18 ^b^	1.05 ± 0.08 ^a^	0.49 ± 0.06 ^a^	1.55 ± 0.02	47.7 ± 1.30	12.8 ± 1.74
	T3	2.11 ± 0.14 ^ab^	1.20 ± 0.13 ^b^	0.69 ± 0.09	3.25 ± 1.08	8.80 ± 0.60	21.4 ± 1.54
Significance		*	*	*	NS	NS	*
Marcona	T1	1.80 ± 0.08	1.20 ± 0.08	0.84 ± 0.05	1.41 ± 0.09 ^a^	54.6 ± 2.80 ^a^	10.2 ± 0.82
	T2	1.70 ± 0.12	1.14 ± 0.11	0.75 ± 0.07	1.63 ± 0.09 ^a^	50.3 ± 0.50 ^a^	11.8 ± 1.74
	T3	1.74 ± 0.05	1.17 ± 0.05	0.62 ± 0.06	3.71 ± 0.15	9.30 ± 1.10	21.2 ± 5.33
Significance		NS	NS	*	NS	*	*

Statistical differences among samples were tested by the Kruskal–Wallis test at the 5% level of significance (NS: not significant. *: significant). For the samples that showed statistical differences, a common letter indicates that samples are not significantly different based on the pair-wise Games–Howell post-hoc test at the 5% level of significance.

**Table 2:** Phenolic compound concentrations (mg/kg) of five almond cultivars submitted to in vitro digestion-fermentation.

	Guara	Vairo	Marta	Marinada	Marcona	Significance
(+)-Catechin	104 ± 48.0	97.7 ± 55.6	73.4 ± 53.1	67.7 ± 63.3	83.0 ± 48.2	NS
(-)-Epicatechin	140 ± 65.8 ^ab^	90.1 ± 35.7 ^acd^	66.4 ± 18.9 ^bce^	38.2 ± 15.3 ^f^	49.0 ± 17.1 ^def^	*
(-)-Epigallocatechin	49.9 ± 9.33 ^abcd^	61.6 ± 9.71 ^aef^	43.2 ± 14.5 ^begh^	33.4 ± 15.8 ^cgi^	52.0 ± 15.2 ^dfhi^	*
Ferulic acid	0.002 ± 0 ^abcd^	0.005 ± 0.003 ^aefg^	0.002 ± 0 ^behi^	0.002 ± 0.001 ^cfhj^	0.002 ± 0 ^dgij^	*
Kaempferol	0.006 ± 0.002	0.005 ± 0.001	0.006 ± 0.002	0.004 ± 0.001	0.008 ± 0.003	NS
Naringenin	0.003 ± 0.001	0.004 ± 0	0.003 ± 0.001	0.003 ± 0.001	0.003 ± 0.001	NS
Naringin	0.003 ± 0.002	0.003 ± 0.001	0.002 ± 0.001	0.002 ± 0	0.003 ± 0.001	NS
*p*-Coumaric acid	0.002 ± 0.001 ^abcd^	0.004 ± 0.003 ^aefg^	0.002 ± 0.002 ^behi^	0.001 ± 0 ^cfhj^	0.006 ± 0.006 ^dgij^	*
Quercetin	54.4 ± 16.2	61.8 ± 8.64	51.9 ± 10.5	51.8 ± 15.0	53.6 ± 13.8	NS
Rutin	84.0 ± 11.6 ^abcd^	80.7 ± 7.86 ^aefg^	75.6 ± 5.03 ^behi^	76.7 ± 2.28 ^cfh^	81.9 ± 3.63 ^dgi^	*
3,4-dihydroxyphenylacetic acid	38.4 ± 14.3	35.4 ± 10.3	31.5 ± 13.4	23.7 ± 13.5	28.7 ± 5.84	NS
3-(3,4-dihydroxyphenyl)propionic acid	269 ± 106 ^ab^	146 ± 73.4 ^acde^	84.3 ± 66.7 ^cfg^	87.8 ± 42.0 ^dfh^	161 ± 95.6 ^begh^	*
3-(3-hydroxyphenyl)propionic acid	21.0 ± 7.28 ^abcd^	4.97 ± 11.6 ^aefg^	9.20 ± 8.17 ^behi^	11.7 ± 12.8 ^cfhj^	19.1 ± 11.7 ^dgij^	*
5-(3′,4′-dihydroxyphenyl)-γ-valerolactone	54.6 ± 57.0 ^ab^	183 ± 157 ^acde^	274 ± 115 ^cfg^	240 ± 113 ^dfh^	213 ± 134 ^begh^	*
Phenol	9.45 ± 2.79	8.41 ± 1.72	9.04 ± 4.11	10.6 ± 4.38	9.79 ± 4.42	NS
Phloroglucinol	207 ± 80.2 ^abcd^	259 ± 56.2 ^ae^	199 ± 34.4 ^befg^	168 ± 46.7 ^cfh^	186 ± 35.5 ^dgh^	*
Urolithin A	0.022 ± 0.011	0.026 ± 0.009	0.021 ± 0.008	0.017 ± 0.006	0.025 ± 0.016	NS
Urolithin B	0.002 ± 0 ^abc^	0.002 ± 0.001 ^adef^	0.001 ± 0 ^d^	0.002 ± 0 ^be^	0.004 ± 0.002 ^cf^	*
Total	1055 ± 39.2	1027 ± 40.9	921 ± 113	814 ± 24.0	944 ± 24.3	NS

Statistical differences among samples were tested by the Kruskal–Wallis test at the 5% level of significance (NS: not significant. *: significant). For the samples that showed statistical differences, a common letter indicates that samples are not significantly different based on the pair-wise Games–Howell post-hoc test at the 5% level of significance.

**Table 3:** Phenolic compound concentrations (mg/kg) of almonds collected at three harvest times and submitted to an in vitro digestion-fermentation.

	T1	T2	T3	Significance
(+)-Catechin	97.3 ± 55.6	71.5 ± 53.9	85.6 ± 50.5	NS
(-)-Epicatechin	97.3 ± 56.9	78.7 ± 59.2	53.6 ± 18.7	NS
(-)-Epigallocatechin	54.1 ± 18.5 ^ab^	47.6 ± 13.3 ^ac^	40.2 ± 12.11 ^bc^	*
Ferulic acid	0.003 ± 0.002	0.002 ± 0.001	0.003 ± 0.002	NS
Kaempferol	0.006 ± 0.003	0.005 ± 0.002	0.007 ± 0.002	NS
Naringenin	0.003 ± 0.001 ^a^	0.003 ± 0.001 ^ab^	0.004 ± 0.001 ^b^	*
Naringin	0.002 ± 0.001	0.003 ± 0.001	0.003 ± 0.001	NS
*p*-Coumaric acid	0.004 ± 0.005	0.002 ± 0.002	0.003 ± 0.002	NS
Quercetin	67.9 ± 5.92	48.0 ± 9.78 ^a^	48.2 ± 10.3 ^a^	*
Rutin	81.8 ± 10.0	81.1 ± 5.24	76.6 ± 4.77	NS
3,4-dihydroxyphenylacetic acid	36.6 ± 12.4	32.6 ± 7.41	26.5 ± 14.7	NS
3-(3,4-dihydroxyphenyl)propionic acid	162 ± 99.3	139 ± 70.3	142 ± 133	NS
3-(3-hydroxyphenyl)propionic acid	16.2 ± 12.6	10.7 ± 8.78	13.6 ± 13.2	NS
5-(3′,4′-dihydroxyphenyl)-γ-valerolactone	214 ± 117	180 ± 138	185 ± 163	NS
Phenol	12.5 ± 2.86	8.77 ± 2.86 ^a^	7.08 ± 2.57 ^a^	*
Phloroglucinol	220 ± 69.2	204.7 ± 47.1	186 ± 59.3	NS
Urolithin A	0.032 ± 0.011	0.018 ± 0.006 ^a^	0.016 ± 0.005 ^a^	*
Urolithin B	0.002 ± 0.002	0.002 ± 0.001	0.002 ± 0.001	NS
Total	1073 ± 49.4	921 ± 72.1	863 ± 159	NS

Statistical differences among samples were tested by the Kruskal–Wallis test at the 5% level of significance (NS: not significant. *: significant). For the samples that showed statistical differences, a common letter indicates that samples are not significantly different based on the pair-wise Games–Howell post-hoc test at the 5% level of significance.

**Table 4:** SCFAs, lactic and succinic acids concentrations (mM) of five almond cultivars collected at three harvest times and submitted to in vitro digestion-fermentation.

Harvest Time	Acetic Acid	Propionic Acid	Butyric Acid	Lactic Acid	Succinic Acid	Total SCFAs
T1	12.0 ± 1.62	1.80 ± 0.38 ^ab^	0.44 ± 0.25 ^a^	7.13 ± 0.65	12.2 ± 0.22 ^ab^	14.3 ± 2.22
T2	12.9 ± 2.61	3.51 ± 2.17 ^ac^	0.50 ± 0.31 ^a^	6.89 ± 0.58	8.62 ± 4.67 ^ac^	15.6 ± 4.65
T3	14.5 ± 6.12	3.82 ± 3.05 ^bc^	2.35 ± 1.92	7.36 ± 1.00	12.2 ± 2.64 ^bc^	16.9 ± 10.9
Significance	NS	*	*	NS	*	NS
**Cultivar**	**Acetic acid**	**Propionic acid**	**Butyric acid**	**Lactic acid**	**Succinic acid**	**Total SCFAs**
Guara	19.5 ± 5.16 ^abc^	6.16 ± 3.2 ^abcd^	2.48 ± 2.29 ^abcd^	6.07 ± 0.19 ^a^	7.50 ± 3.76 ^abcd^	28.2 ± 10.4 ^abcd^
Vairo	12.6 ± 0.92 ^ade^	2.21 ± 0.57 ^aefg^	0.97 ± 1.00 ^aefg^	7.58 ± 0.10 ^bcd^	12.2 ± 0.62 ^aefg^	15.7 ± 2.42 ^aefg^
Marta	12.1 ± 0.38 ^bd^	2.25 ± 0.44 ^behi^	1.22 ± 1.35 ^behi^	7.49 ± 0.33 ^bef^	12.8 ± 0.56 ^behi^	15.6 ± 1.46 ^beh^
Marinada	11.1 ± 0.08 ^ce^	2.97 ± 1.94 ^cfhj^	0.33 ± 0.05 ^cfhj^	7.23 ± 0.53 ^ceg^	9.54 ± 5.10 ^cfhj^	14.4 ± 2.03 ^cfhi^
Marcona	10.6 ± 0.09	1.63 ± 0.18 ^dgij^	0.48 ± 0.24 ^dgij^	7.26 ± 1.09 ^adfg^	13.0 ± 0.74 ^dgij^	12.7 ± 0.36 ^dgi^
Significance	*	*	*	*	*	*

Statistical differences among samples were tested by the Kruskal–Wallis test at the *5*% level of significance (NS: not significant. *: significant). For the samples that showed statistical differences, a common letter indicates that samples are not significantly different based on the pair-wise Games–Howell post-hoc test at the 5% level of significance.

## Data Availability

Data are available to other researchers upon request to the corresponding authors (J.Á.R.-H.).

## Supplementary Materials

The following supporting information can be downloaded at: https://www.mdpi.com/article/10.3390/antiox13010084/s1, Table S1: Morphological measurements of almonds collected at three harvest times and subjected to in vitro digestion-fermentation; Table S2: Morphological measurements of five almond cultivars subjected to in vitro digestion-fermentation; Table S3: Ash, moisture and protein content of five almond cultivars subjected to in vitro digestion-fermentation; Table S4: Ash, moisture and protein content of almonds collected at three harvest times and subjected to in vitro digestion-fermentation; Table S5: Total antioxidant capacity of almonds collected at three harvest times and subjected to in vitro digestion-fermentation; Table S6: Total antioxidant capacity of five almond cultivars subjected to in vitro digestion-fermentation.
