# Supplementary material for: Cultivar and Harvest Time of Almonds Affect Their Antioxidant and Nutritional Profile through Gut Microbiota Modifications
Source: Antioxidants (Basel). 2024 Jan 9;13(1):84. doi: 10.3390/antiox13010084 (PMC10812595; doi:10.3390/antiox13010084)
Supplement: Supplementary file 1 [file antioxidants-13-00084-s001.zip › antioxidants-2801337-supplementary.pdf]

Supplementary Materials

Cultivar and harvest time of almonds modify the antioxidant and nutritional profile of almonds through gut microbiota modifications

Adriana Delgado-Osorio [1], Beatriz Navajas-Porras [1], Sergio Pérez-Burillo [1], Daniel Hinojosa-Nogueira [1], Ángela Toledano-Marín [1], Silvia Pastoriza de la Cueva [1], Oleg Paliy [2] and José Ángel Rufián-Henares [1,3,*]

[1] Departamento de Nutrición y Bromatología, Instituto de Nutrición y Tecnología de los Alimentos, Centro de Investigación Biomédica, Universidad de Granada. Av. del Hospicio, s/n, 18012 Granada, Spain; adrianadelgado@ugr.es (A.D.O.), beatriznavajas@ugr.es (B.N.P.), spburillo@ugr.es (S.P.B.), dhinojosa@ugr.es (D.H.), antolemarin@correo.ugr.es (A.T.M.), spdelacueva@ugr.es (S.P.), jarufian@ugr.es (J.A.R.H.)

[2] Department of Biochemistry and Molecular Biology, Boonshoft School of Medicine, Wright State University, Dayton, Ohio, USA; oleg.paliy@wright.edu (O.P.)

[3] Instituto de Investigación Biosanitaria ibs.GRANADA, Universidad de Granada. Avda. de Madrid 15, 2a Planta, 18012 Granada Granada, Spain; jarufian@ugr.es

*Correspondence: jarufian@ugr.es;

Table S1. Morphological measurements of almonds collected at three harvest times and submitted to *in vitro* digestion-fermentation.

Harvest Time	Length (cm)	Width (cm)	Thickness (cm)
**T1**	2.18 ± 0.26	1.09 ± 0.12[a]	0.71 ± 0.11[a]
**T2**	2.19 ± 0.30	1.17 ± 0.11	0.68 ± 0.09[a]
**T3**	2.15 ± 0.25	1.08 ± 0.13[a]	0.46 ± 0.11
**Significance**	NS	*	*

Statistical differences among samples were tested by the Kruskal-Wallis test at the 5% level of significance (NS: not significant. *: significant). For the samples that showed statistical differences, a common letter indicates that samples are not significantly different based on the pair-wise Games-Howell *post hoc* test at the 5% level of significance.

Table S2. Morphological measurements of five almond cultivars submitted to *in vitro* digestion-fermentation.

Cultivar	Length (cm)	Width (cm)	Thickness (cm)
**Guara**	2.46 ± 0.11	1.17 ± 0.13[abc]	0.60 ± 0.16[ab]
**Vairo**	2.23 ± 0.11[ab]	1.09 ± 0.10[adef]	0.49 ± 0.11
**Marta**	2.29 ± 0.12[a]	1.01 ± 0.12[d]	0.62 ± 0.16[ac]
**Marinada**	2.15 ± 0.17[b]	1.11 ± 0.12[beg]	0.63 ± 0.12[bc]
**Marcona**	1.75 ± 0.09	1.17 ± 0.08[cfg]	0.74 ± 0.11
**Significance**	*	*	*

Statistical differences among samples were tested by the Kruskal-Wallis test at the 5% level of significance (NS: not significant. *: significant). For the samples that showed statistical differences, a common letter indicates that samples are not significantly different based on the pair-wise Games-Howell *post hoc* test at the 5% level of significance.

Table S3. Ash, moisture and protein content of five almond cultivars submitted to *in vitro* digestion-fermentation.

Cultivar	Moisture (%)	Protein (%)	Ash (%)
**Guara**	40.01 ± 26.72	17.20 ± 7.33	2.78 ± 2.15
**Vairo**	37.96 ± 26.06	17.99 ± 5.16	2.30 ± 1.04
**Marta**	42.84 ± 28.69	15.71 ± 6.86	2.52 ± 1.61
**Marinada**	44.37 ± 30.43	14.58 ± 6.98	1.98 ± 1.11
**Marcona**	38.05 ± 22.40	15.27 ± 6.25	2.25 ± 1.14
**Significance**	NS	NS	NS

Statistical differences among samples were tested by the Kruskal-Wallis test at the 5% level of significance (NS: not significant. *: significant). For the samples that showed statistical differences, a common letter indicates that samples are not significantly different based on the pair-wise Games-Howell *post hoc* test at the 5% level of significance.

Table S4. Ash, moisture and protein content of almonds collected at three harvest times and submitted to *in vitro* digestion-fermentation.

Harvest Time	Moisture (%)	Protein (%)	Ash (%)
**T1**	65.26 ± 7.55	11.81 ± 3.72[a]	1.38 ± 0.17
**T2**	49.16 ± 3.74	12.86 ± 2.15[a]	1.57 ± 0.14
**T3**	7.52 ± 1.43	23.78 ± 2.97	4.14 ± 0.98
**Significance**	*	*	*

Statistical differences among samples were tested by the Kruskal-Wallis test at the 5% level of significance (NS: not significant. *: significant). For the samples that showed statistical differences, a common letter indicates that samples are not significantly different based on the pair-wise Games-Howell *post hoc* test at the 5% level of significance.

Table S5. Total antioxidant capacity of almonds collected at three harvest times and submitted to *in vitro* digestion-fermentation.

Harvest Time	$TEAC_{FRAP}$ (mmol Trolox equivalents/kg)	$TEAC_{DPPH}$ (mmol Trolox equivalents/kg)	$TEAC_{ABTS}$ (mmol Trolox equivalents/kg)	Folin-Ciocalteu (mg gallic acid equivalents/kg)
**T1**	248 ± 55.1	220 ± 14.97	1312 ± 116	44640 ± 3509
**T2**	235 ± 17.08	217 ± 17.59	1270 ± 118	48693 ± 2630[a]
**T3**	237 ± 28.16	215 ± 25.48	1283 ± 115	51060 ± 5531[a]
**Significance**	NS	NS	NS	*

Statistical differences among samples were tested by the Kruskal-Wallis test at the *5%* level of significance (NS: not significant. *: significant). For the samples that showed statistical differences, a common letter indicates that samples are not significantly different based on the pair-wise Games-Howell *post hoc* test at the *5%* level of significance.

Table S6. Total antioxidant capacity of five almond cultivars submitted to *in vitro* digestion-fermentation.

Cultivar	$TEAC_{FRAP}$ (mmol Trolox equivalents/kg)	$TEAC_{DPPH}$ (mmol Trolox equivalents/kg)	$TEAC_{ABTS}$ (mmol Trolox equivalents/kg)	Folin-Ciocalteu (mg gallic acid equivalents/kg)
**Guara**	266 ± 38.38[abc]	231 ± 12.17[abcd]	1280 ± 107	50683 ± 5585
**Vairo**	200 ± 23.92[de]	20 ± 14.24[aefg]	1295 ± 177	45913 ± 3079
**Marta**	241 ± 26.71[adfg]	217. ± 18.71[behi]	1306 ± 47	50986 ± 3883
**Marinada**	264 ± 24.52[bfh]	200 ± 20.55[cfhj]	1311 ± 96	47672 ± 2171
**Marcona**	230 ± 25.81[cegh]	230 ± 12.49[dgij]	1250 ± 133	45403 ± 6220
**Significance**	*	*	NS	NS

Statistical differences among samples were tested by the Kruskal-Wallis test at the *5%* level of significance (NS: not significant. *: significant). For the samples that showed statistical differences, a common letter indicates that samples are not significantly different based on the pair-wise Games-Howell *post hoc* test at the *5%* level of significance.
